# Temporal Trends in Stomach and Colorectal Cancer Mortality by Racial Groups in Brazil (2000–2023): A Longitudinal Ecological Study

**DOI:** 10.3390/ijerph22020208

**Published:** 2025-01-31

**Authors:** Karina Cardoso Meira, Raphael Mendonça Guimarães, Nathalia Sernizon Guimarães

**Affiliations:** 1 Department of Pharmaceutical Sciences, Federal University of São Paulo, Diadema 09913-030, SP, Brazil; 2 Oswaldo Cruz Foundation, National School of Public Health, Rio de Janeiro 21040-900, RJ, Brazil; raphael.guimaraes@fiocruz.br; 3 Department of Nutrition, School of Nursing, Universidade Federal de Minas Gerais, Belo Horizonte 30130-100, MG, Brazil; nasernizon@gmail.com

**Keywords:** cancer, mortality, racial disparities, time-series, Brazil

## Abstract

This longitudinal ecological study analyzed racial disparities in mortality trends for stomach cancer (SC) and colorectal cancer (CRC) in Brazil (2000 to 2023) stratified by sex. Data from the National Mortality Information System were analyzed for individuals aged 25 to 80. Self-reported race/skin color followed the Brazilian Institute of Geography and Statistics classification: White (White group) and Black or Brown/multiracial (Black group). Age-standardized mortality rates used the world population as a reference, and Prais–Winsten autoregression calculated trends. SC mortality rates declined for both sexes and racial groups, with a greater reduction in the Annual Percent Change (APC) among Whiteindividuals. Conversely, CRC mortality rates increased, with the Black group showing a higher percentage increase in APC. Despite progress in reducing SC mortality, disparities persist, particularly for CRC, where Black populations experience worse outcomes. Higher SC and CRC mortality rates were observed among White individuals, but the trends highlight the growing burden of CRC in Black populations. These findings emphasize the urgent need to address racial disparities in cancer outcomes, as they remain a critical public health challenge despite advancements in healthcare access and disease control in Brazil.

## 1. Introduction

Cancer remains a leading cause of global illness and death, with an estimated 20 million new cases and approximately 10 million deaths projected for 2022 [1]. The cancer burden varies significantly across regions, disproportionately impacting low- and middle-income countries due to limited resources and restricted access to comprehensive oncological care [2,3,4,5]. In these regions, late-stage diagnoses and inadequate access to advanced treatments contribute to higher mortality rates [1,2,3,4,5].

Modifiable risk factors such as smoking, excessive alcohol consumption, poor diet, obesity, and physical inactivity play a critical role in cancer development. In countries with a low Human Development Index (HDI), chronic infections also drive infection-associated cancers, including stomach, cervical, and liver cancers [3,4,5,6,7,8]. Conversely, high-HDI countries face increasing rates of lifestyle-related cancers, such as colorectal cancer, linked to shifts toward Western diets and sedentary lifestyles [8,9,10,11].

Globally, colorectal cancer (CRC) and stomach cancer (SC) are among the most burdensome gastrointestinal cancers. In 2022, CRC accounted for approximately 1.9 million new cases and 935,000 deaths, ranking as the second most common cancer and the fourth leading cause of cancer-related deaths worldwide. Similarly, SC was responsible for about one million new cases and 769,000 deaths, making it the sixth most common cancer and the fourth leading cause of cancer mortality [1].

The World Health Organization (WHO) reports the highest CRC incidence rates in high-income countries, with Southern and Northern Europe exceeding 20.0 new cases per 100,000 men and 15.0 per 100,000 women. In contrast, Central Africa and South–Central Asia report the lowest rates, with 3.0 new cases per 100,000 men and 2.0 per 100,000 women. Mortality rates, however, vary less, with high-income countries reporting 13.1 deaths per 100,000 and low- and middle-income countries reporting 4.7 deaths per 100,000, a rate ratio of 2.8 [1].

A similar pattern exists for SC. East Asia reports the highest incidence rates, with 32.5 new cases per 100,000 men and 13.2 per 100,000 women, while Southern Africa has the lowest rates, with 4.7 and 2.4 new cases per 100,000 men and women, respectively. Mortality rates are comparable between high- and low-income countries, at 3.9 and 3.5 deaths per 100,000, respectively [1].

In Brazil, the estimated cancer burden for 2022–2025 reflects global trends. CRC ranks as the third most common cancer, with 21,970 new cases annually, surpassed only by breast and prostate cancers. SC ranks fifth, with 17,010 new cases annually, following breast, prostate, colorectal, and lung cancers [12].

Disparities in cancer incidence and mortality based on the HDI are well documented. High-HDI countries, having undergone a “cancer transition”, report a higher CRC incidence but a lower SC incidence. Improved access to timely oncological care has reduced mortality rates for both cancers. In Brazil, regional disparities follow similar trends: high-income regions report rising CRC incidence and mortality rates, while SC incidence and mortality rates are declining. Conversely, low- and middle-income regions exhibit the opposite pattern, highlighting socioeconomic inequalities [12,13].

Globally, cancer mortality is nearly twice as high as its incidence, with death rates 43% higher in men than in women (120.8 vs. 84.2 per 100,000, respectively). This disparity is also seen in CRC and SC. In 2020, CRC mortality was 1.52 times higher in men than in women (9.9 vs. 6.5 deaths per 100,000). Similarly, SC mortality in men was 2.20 times higher than in women (8.6 vs. 3.9 deaths per 100,000) [1]. These differences are attributed to higher male exposure to risk factors and less frequent use of healthcare services, leading to delayed diagnoses and lower survival rates [14,15,16].

Social and racial disparities further exacerbate inequities in cancer outcomes. In the United States, Black populations face higher CRC mortality and lower survival rates compared to White populations. Between 2000 and 2017, CRC mortality was 38% higher among Black individuals than White individuals (19.0 vs. 13.8 per 100,000). Five-year relative survival rates were also lower for Black patients (57.6% vs. 69.1%). Notably, survival rates improved for White patients from 1992–2002 to 2003–2013, but no improvement was observed for Black patients [17].

Disparities are also evident in non-cardia gastric cancer. Mortality rates are two to three times higher among Black individuals, Hispanics, and Asians/Pacific Islanders compared to non-Hispanic Whites. These differences persist across all age groups and disease stages [18,19]. Marginalized racial and ethnic populations consistently face higher risks of diagnosis and death from this condition [19].

In Brazil, Black and Indigenous populations are more likely to receive late-stage diagnoses compared to White individuals. These disparities reflect systemic barriers to accessing preventive and curative care, even within Brazil’s universal health system [20,21,22,23,24,25]. Structural racism, rooted in the country’s history of slavery, continues to shape social, economic, and health inequities, disproportionately affecting Afro-descendant populations [20,26,27,28,29,30,31].

Despite these challenges, few Brazilian studies have analyzed racial disparities in national mortality rates. This gap is largely due to the lack of population estimates stratified by race/skin color and age group [2,25,31,32]. Official statistics often disaggregate data only by sex, limiting insights into racial disparities in health outcomes [2].

This study aims to address these gaps by examining racial disparities, stratified by sex, in mortality trends for stomach and colorectal cancers in Brazil from 2000 to 2023.

## 2. Materials and Methods

### 2.1. Study Design and Location

This ecological study adhered to the Guidelines for Accurate and Transparent Health Estimates Reporting (GATHER) statement [33].

The 2022 Census estimated Brazil’s population at 207.8 million. Of this total, approximately 92.1 million people (45.3%) identified as multiracial or Brown (“pardos”). Another 88.2 million (43.5%) identified as White, while 20.6 million (10.2%) identified as Black (“pretos”). The Brazilian Institute of Geography and Statistics (IBGE) classifies individuals who self-identify as “Black” (“preto”) or “multiracial or Brown” (“pardo”) collectively as “Black” (“negro”) [34,35].

### 2.2. Data Source

Mortality data were obtained from the Mortality Information System (SIM) through the Informatics Department of the Brazilian Unified Health System (SUS) [30]. Population estimates by race/skin color were retrieved from The Portrait of Gender and Race Inequalities, a dataset published by the Economic Research Institute (IPEA). This dataset provides disaggregated data by sex and race/skin color for three categories: White, Black, and Brown [36].

We analyzed mortality data by race/skin color (White and Black) for individuals aged 25 years and older. Age groups were stratified into 25–29, 30–44, 45–59, and 60+ years. Individuals under 25 years of age were excluded from the analysis for two primary reasons: (i) younger age groups had excessive zero counts, reducing statistical reliability, and (ii) higher incidence and mortality rates for colorectal cancer (CRC) and stomach cancer (SC) are generally observed from the third decade of life onward, reflecting cumulative exposure to risk factors [1,2,3,4,5].

### 2.3. Correction of Death Records

While the quality of data in the SIM has improved, a substantial portion of records remain classified under ill-defined causes or garbage codes [32]. This limits the reliability of temporal trend analyses. To address this, the methodology developed by the World Health Organization (WHO) [33], as adopted in previous Brazilian studies [13,37,38,39,40,41], was employed to correct death records.

Corrections for stomach cancer (SC) and colorectal cancer (CRC) were performed separately, stratified by sex and race/skin color, in three steps:

(i) Redistribution of ill-defined causes: Half of deaths classified as ill-defined (R00–R99) were redistributed by year and age group using the WHO methodology [33];
(ii) Redistribution of incomplete diagnoses: Deaths with incomplete cancer diagnoses (e.g., general cancer C76–C80 and C97, or gastrointestinal tract cancer C26) were proportionally redistributed by year and age group [33];
(iii) Data integration: The results from steps 1 and 2 were combined with cancer deaths recorded in the SIM/DATASUS to generate the corrected dataset [13,37,38,39].

Microdata, initially in the .dbc format, were converted to .dbf using Tabwin version 4.15 for Windows, provided by the Brazilian Ministry of Health. Data from 2000 to 2023 were processed, selecting only ICD-10 codes for stomach and colorectal cancers for individuals aged 25 years or older [35].

### 2.4. Data Analysis

After correcting the death records, age-specific mortality rates were calculated for the age groups 25–29, 30–44, 45–59, and 60 years and older. These rates were standardized using the direct method, with the World Health Organization’s standard population for 2000–2025 serving as the reference [42]. Mortality rates were expressed per 100,000 individuals and stratified by sex and race/skin color for stomach and colorectal cancers over the period 2000–2023.

Mortality rates were also calculated for six four-year intervals: 2000–2003, 2004–2007, 2008–2011, 2012–2015, 2016–2019, and 2020–2023. Statistical comparisons were conducted using Student’s *t*-test and analysis of variance (ANOVA), following verification of normal distribution with the Shapiro–Wilk and Kolmogorov–Smirnov tests.

Temporal trends were visualized through line graphs. To reduce random fluctuations in the data, a three-year moving average was applied to smooth the mortality rates [42,43].

### 2.5. Time Trend Analysis Using the Prais–Winsten Method

The study utilized the Prais–Winsten regression model, a generalized least squares regression method designed to account for first-order serial autocorrelation in time-ordered data. Mortality rates, log-transformed to base 10, were employed as the dependent variable, while the year of death served as the independent variable. This approach corrected for autocorrelation, thereby enhancing the robustness and reliability of the trend estimates. The analysis was based on the following equations [43,44]:

log10(mortality ratet) = β_0_ + β_1_(Year_t_) + ε_t_ (1)

where:

ε_t_ = p·ε_t_ − 1 + u_t_

In this formulation, log10 (mortality rate_t_) represents the base-10 logarithm of the mortality rate in year t, β_0_ denotes the intercept, β1 captures the annual change in the logarithmic scale, and ε_t_ indicates the residual (or error) at time t.

The Prais–Winsten model assumes that residuals (ε_t_) follow a process where ε_t_ = p·ε_t_ − 1 + u_t_, with p representing the correlation between consecutive errors, and u_t_ denoting uncorrelated random noise.

The regression coefficient β_1_ derived from the log-transformed model was subsequently used to calculate the Annual Percent Change (APC), a measure of yearly variation in mortality rates. The APC was computed using the following formula:

APC = (−1 + 10^β_1_^)·100 (2)

To construct 95% confidence intervals (CIs) for the APC, the study first determined the lower and upper bounds of β_1_ as follows:

β_1_^lower^ = β_1_−t_n−2_·SE(β_1_) (3)

β_1_^upper^ = β_1_ + t_n−2_·SE(β_1_) (4)

Here, t(n−2) r refers to the critical value of the t-distribution with n−2 degrees of freedom (α = 0.05) and SE(β_1_) represents the standard error of the coefficient. These bounds were subsequently transformed to the APC scale using the same base-10 transformation:

APC^lower^ = (−1 + 10^β_1_ lower^)·100 (5)

APC^upper^= (−1 + 10^β^_1_ ^upper^)·100 (6)

All analyses were performed in Python using the Spyder 5.5.1 Integrated Development Environment (IDE). Data analysis and visualization were conducted with the assistance of libraries such as matplotlib, math, numpy, pandas, statsmodels, and scipy.stats. Statistical significance was determined using a threshold of *p* < 0.05.

### 2.6. Ethical Aspects

The researchers used publicly available data from the SIM/DATASUS, which excludes any identifiable information about individual subjects. As a result, the study did not require approval from a research ethics committee, in accordance with Article 1 of CNS Resolution No. 510, issued on 7 April 2016 [45].

## 3. Results

### 3.1. Descriptive Analysis

In Brazil, CCR caused 642,713 deaths during the study period. Men accounted for most of these deaths (56.05%; n = 360,219), while women represented 43.95% (n = 282,494). SC caused 302,620 deaths, with men experiencing a higher proportion (64.49%) compared to women (35.51%). More than 98% of these deaths occurred among individuals identified as White or Black (including Brown).

**Figure 1:**
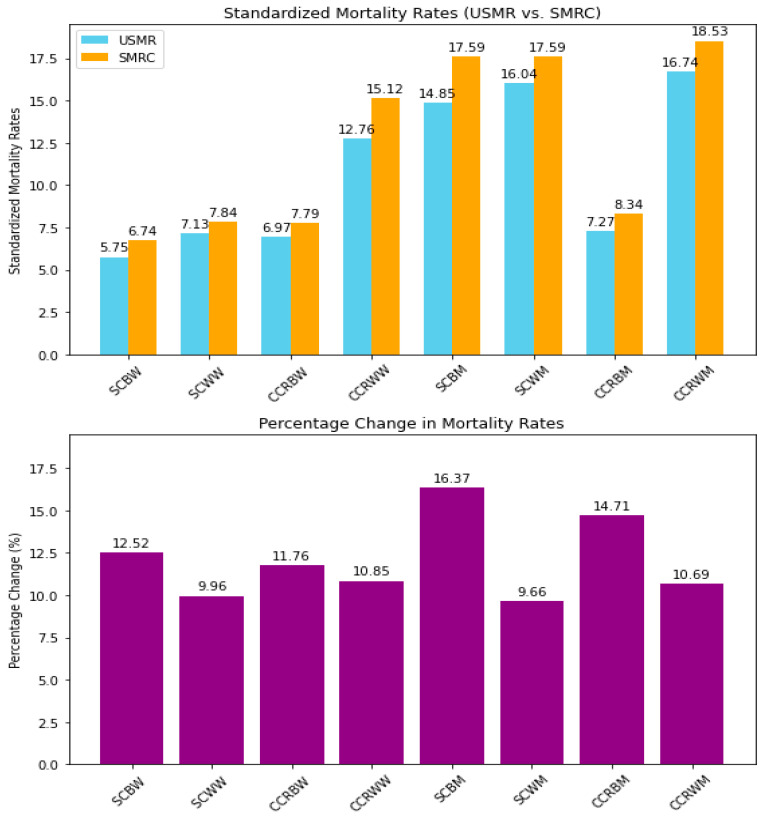
Uncorrected and corrected standardized mortality rates per 100,000 for stomach cancer and colorectal cancer in Brazil (2000 to 2023). Note: USMR—uncorrected standardized mortality rate; SMRC—standardized mortality rate with correction; SCWW—stomach cancer in White women; SCBW—stomach cancer in Black women; CCRBW—colorectal cancer in Black women; CCRWW—colorectal cancer in White women; SCWM—stomach cancer in White men; SCBM—stomach cancer in Black men; CCRWM—colorectal cancer in White men; CCRBM—colorectal cancer in Black men. Source: Mortality Information System (SIM/SUS)|Institute of Applied Economic Research (Instituto de Pesquisas Econômicas e Aplicadas—IPEA).

Adjusting for under-reporting revealed a greater increase in standardized mortality rates for SC among Black women. Their rates rose by 12.52% (from 5.75 to 6.47 deaths per 100,000), compared to a 9.96% rise among White women (from 7.13 to 7.84 deaths per 100,000) (Figure 1, Table S1). For CCR, Black women showed an 11.76% increase (from 6.97 to 7.79 deaths per 100,000), whereas White women had a 10.85% rise (from 13.64 to 15.12 deaths per 100,000) (Figure 1, Table S1).

Among men, Black individuals showed the highest percentage increases. Black men experienced a 16.37% rise in corrected SC mortality rates (from 12.76 to 14.85 deaths per 100,000) and a 14.71% increase for colorectal cancer (from 7.27 to 8.34 deaths per 100,000) (Figure 1, Table S1).

White individuals consistently had higher mortality rates for both cancers. Between 2000 and 2023, SC mortality rates among White women were 1.22 times higher than those among Black women (7.84 vs. 6.42 deaths per 100,000). Among men, the disparity was smaller; White men had rates 1.19 times higher than Black men (17.59 vs. 14.85 deaths per 100,000). For CCR, the differences were more pronounced, with rates 1.94 times higher in White women (15.12 vs. 7.79 deaths per 100,000) and 2.22 times higher in White men (18.53 vs. 8.34 deaths per 100,000) (Figure 1, Table S1).

Men consistently showed higher mortality rates than women within each racial group. However, White women had higher mortality rates than Black men in several instances (Figure 1, Table S1). Across all four-year periods, White women maintained higher mortality rates than Black women for both cancers, with statistically significant differences (*p* < 0.05) (Table 1). Among men, a similar trend appeared, except during the final four-year period for SC, where the difference was not statistically significant (*p* = 0.979) (Table 2).

**Figure 2:**
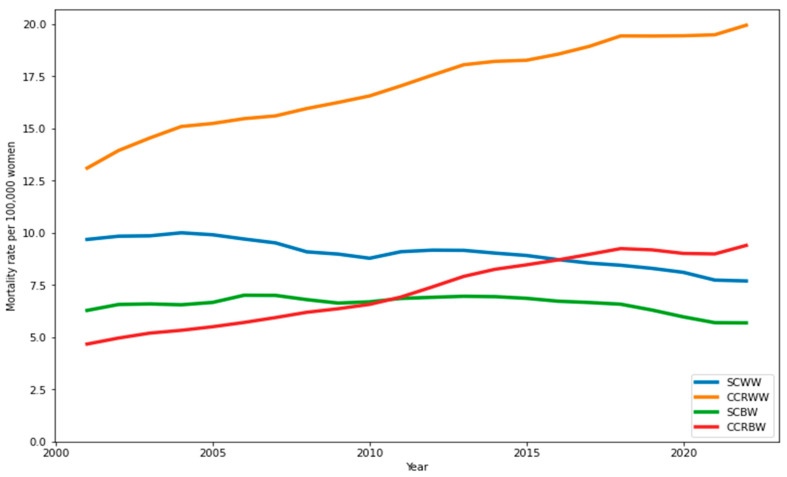
Mortality rates smoothed by three-year moving averages per 100,000 women in Brazil (2000 to 2023). Note: SCWW—stomach cancer in White women; SCBW—stomach cancer in Black women; CCRBW—colorectal cancer in Black women; CCRWW—colorectal cancer in White women. Source: Mortality Information System (SIM/SUS)|Institute of Applied Economic Research (Instituto de Pesquisas Econômicas e Aplicadas—IPEA).

**Figure 3:**
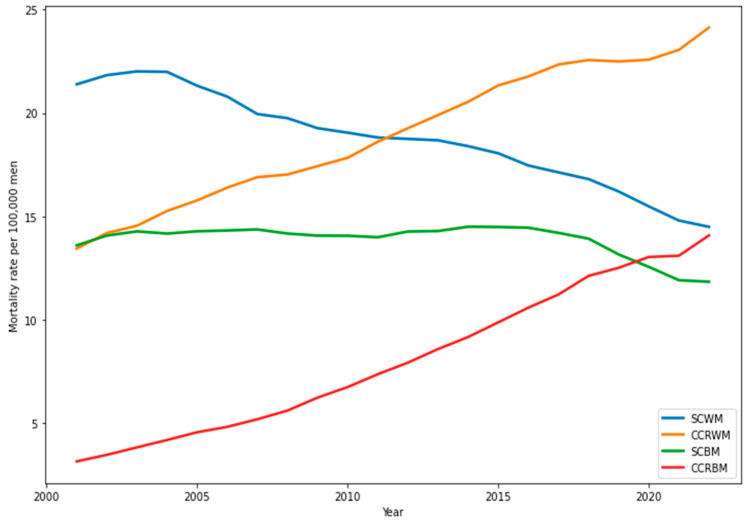
Mortality rates smoothed by three-year moving averages per 100,000 men in Brazil (2000 to 2023). Note: SCWM—stomach cancer in White men; SCBM—stomach cancer in Black men; CCRWM—colorectal cancer in White men; CCRBM—colorectal cancer in Black men. Source: Mortality Information System (SIM/SUS)|Institute of Applied Economic Research (Instituto de Pesquisas Econômicas e Aplicadas—IPEA).

Smoothed mortality rates, calculated using three-year moving averages, showed that CCR mortality consistently surpassed stomach cancer mortality among White women throughout the study period. Among Black women, CCR mortality began to exceed SC mortality after 2010 and continued to rise, eventually surpassing SC rates in White women by the end of the study. Both racial groups saw declines in SC mortality, though the decrease was less pronounced than the increase in CCR mortality (Figure 2 and Figure 3). White women consistently experienced reductions in SC mortality across all quadrennial periods (2000–2003 to 2020–2023). Black women showed stable SC mortality rates of around 6.0 deaths per 100,000, while colorectal cancer mortality rates progressively increased for both racial groups (Table 1 and Table 2).

Among White men, CCR mortality rates surpassed SC rates around 2010, reversing earlier trends. For Black men, SC mortality remained higher than CCR mortality until 2018 (Figure 2). While SC mortality among Black men remained stable for most of the study, it decreased toward the end. In contrast, CCR mortality rose sharply, surpassing SC rates by the study’s conclusion. White men showed consistent reductions in SC mortality, while Black men experienced increases until 2016–2019. CCR mortality rates increased steadily for both groups throughout the study (Table 2).

### 3.2. Bivariate Analysis According to Four-Year Period and Age Group

In both cancers studied, mortality rates increased with advancing age among White and Black women. For SC, mortality rates declined across the four-year periods for women aged 30–44 and ≥60 years. Among middle-aged women (45–59 years), this downward trend persisted until 2012–2015. These reductions were statistically significant at the 5% level among Black women across all age groups and among White women in young adults (30–44 years) and the elderly (≥60 years) (Table 3). In contrast, CRC mortality rates increased across all age groups and four-year periods (*p* < 0.05) (Table 3).

Among men, a similar trend was evident. SC mortality rates progressively decreased across all age groups (30–44 years to ≥60 years) over the four-year periods. However, CRC mortality consistently increased across all age groups among Black men, with statistically significant differences (Table 4 and Table 5). Among White men, significant increases in CRC mortality were observed in all age groups except the youngest (25–29 years) (Table 4).

### 3.3. Analysis of Temporal Trends Using Prais–Winsten Regression

Trend analysis showed an annual reduction of 1.20% (95% CI: −1.52, −0.88%) in SC mortality among White women and 1.12% (95% CI: −1.94, −0.29%) among Black women. However, these differences were not statistically significant at the 5% level. In contrast, CRC mortality increased annually at a rate of 3.13% (95% CI: 2.31–3.95%) among Black women, compared to 1.67% (95% CI: 1.39–1.67%) among White women.

**Figure 4:**
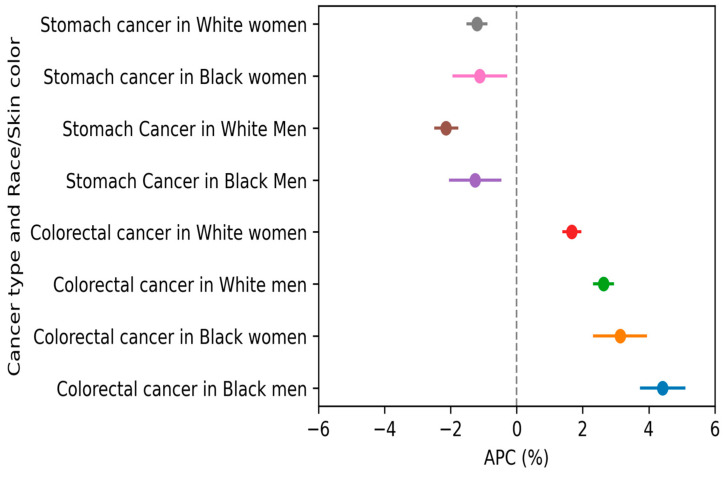
Annual Percent Change for stomach and colorectal cancer by sex and race/skin color in Brazil (2000 to 2023), estimated using Prais–Winsten regression.

Among men, SC mortality decreased more significantly in White men (APC = −2.14%; 95% CI: −2.50, −1.77%) compared to Black men (APC = −1.26%; 95% CI: −2.05, −0.46%). Conversely, CRC mortality increased at a higher annual rate among Black men (APC = 4.42%; 95% CI: 3.73–5.11%) compared to White men (APC = 2.63%; 95% CI: 2.31–2.95%) (Figure 4, Table S2).

Both racial groups exhibited similar trends across age groups. In SC, mortality reductions were observed among White women aged ≥ 30 years, while among Black women, decreases were most pronounced in middle-aged and elderly groups. For CRC, mortality increased across all age groups among Black women except the youngest (*p* = 0.054). Among White women, increases were limited to those aged 25–44 years (Table 5).

Among men, SC mortality trends were similar across racial groups, with reductions observed in most age groups. However, CRC mortality increased in all age groups among Black men, while among White men, no significant increase was observed in the youngest age group (25–29 years) (*p* = 0.895) (Table 6).

## 4. Discussion

Our findings underscore significant disparities between Black and White populations in Brazil concerning the quality of death certificate registration, mortality rates, and temporal trends for stomach and colorectal cancer. These disparities highlight critical inequities that warrant further investigation and intervention.

The Black population demonstrated lower quality in the certification of deaths related to the two cancers examined. This is evidenced by a greater percentage increase in corrected mortality rates compared to uncorrected rates, particularly among Black men. Accurate certification of cancer-related deaths depends on access to medium- and high-complexity healthcare services, which provide essential diagnostic tests for confirmation [25,41,43,44,45,46,47]. Limited access to such services exacerbates these disparities.

Despite Brazil’s universal and free healthcare system, it has proven insufficient to mitigate the historical health inequities experienced by the Black population. These inequities are rooted in structural social disadvantages imposed by systemic racism, which disproportionately expose Black individuals to risk factors and systemic barriers to screening and treatment. Structural racism perpetuates these disparities by shaping broader health-disease processes [24,47,48,49,50,51].

One manifestation of structural racism is spatial segregation, whereby the Black population predominantly resides in urban peripheries. These areas often lack adequate access to medium- and high-complexity healthcare services, which are typically concentrated in city centers [25,41,43,44,45,46,47]. This geographical disparity further hinders timely diagnosis and effective treatment for Black individuals.

In addition, racial discrimination within healthcare services compounds the challenges faced by the Black population. Institutional racism manifested in practices, policies, norms, and organizational structures affects healthcare professionals’ decision-making processes, diagnosis, treatment, and care for Black patients. This form of racism obstructs access to early diagnosis and increases the likelihood of under-reported cases and deaths being recorded as ill-defined causes [20,21,22,23,24,25,46].

SC remains a major public health issue in Brazil, characterized by high mortality rates and an upward trend in areas with socioeconomic vulnerability and inequitable access to healthcare services [20,21,22,23,24,25,46]. In our study, higher mortality rates for SC were observed among the White population across all age groups. These findings align with a Brazilian study conducted between 2000 and 2015 which reported significantly higher SC mortality rates in the White population compared to the Black population (6.69 vs. 5.51, *p* < 0.001) [31].

Regarding differences in mortality rates by sex, previous studies have consistently reported higher incidence and mortality rates for SC among men [1,2,3,12,13,24,31,32]. In our study, mortality rates for SC and CRC were also higher among men, regardless of race or skin color. However, White women exhibited higher mortality rates than Black men, underscoring the racial disparities within Brazil’s morbidity and mortality profile.

We believe this finding reflects the social disadvantages faced by the Black population, which directly influence their health-disease process and life expectancy [12,13,20,21,22,23,24]. Within this context, Black men have a shorter life expectancy compared to White men and women. Black men experience a disproportionate burden of premature mortality, primarily due to violent deaths (homicides) and infectious diseases, which reduces their likelihood of dying from neoplasms or other chronic conditions [26,29,30].

Although the magnitude of SC mortality rates is lower in the Black population compared to the White population, it is important to highlight the high burden of mortality from this neoplasm, particularly among Black men.

We also observed higher mortality rates of CCR among the White population. The age-standardized mortality rate for White women was 1.96 times higher than that for Black women. Over the historical period analyzed, the mortality rates for White women consistently exceeded those observed for Black women.

It is notable that CRC mortality rates among Black women were comparable to SC mortality rates among White women. Among men, the disparity between these rates was even more pronounced. Furthermore, it is important to emphasize that CRC mortality rates among Black men only surpassed SC mortality rates in the later years of the historical series. These findings align with other Brazilian studies which have reported a lower likelihood of CRC-related deaths among Black and Brown individuals compared to White individuals across all regions of Brazil [47].

The trend analysis of cancer mortality rates by age group, stratified by race/skin color and sex, revealed similar patterns for White and Black populations. While SC mortality rates exhibited a general decreasing trend, CRC mortality rates increased over the analyzed period, reflecting a cancer transition characterized by declining cancers associated with infections and rising rates of cancers linked to Westernized lifestyle habits [1,2,3,4,5].

Nonetheless, significant racial differences in mortality patterns were identified. For example, CRC mortality rates consistently exceeded SC mortality rates in White women throughout the study period, whereas among Black women, this inversion occurred only after 2010. Similarly, among White men, the transition between SC and CRC mortality rates was observed in 2010, but for Black men, despite a progressive increase in CRC rates, this inversion only materialized at the end of the historical series.

These racial disparities may partially stem from differences in risk factors and etiologies associated with stomach cancer subtypes, such as cardia and non-cardia cancers. Research indicates that the incidence of non-cardia stomach cancer is higher among Black individuals compared to White individuals, reflecting the adverse living conditions disproportionately experienced by the Black population [50,51,52,53,54]. The subtypes differ in their risk factors, carcinogenic pathways, and morbidity and mortality profiles. Non-cardia gastric cancer is predominantly associated with Helicobacter pylori infection, the consumption of salt-preserved foods, and insufficient fruit and vegetable intake, while cardia gastric cancer is linked to obesity, gastroesophageal reflux disease, and Barrett’s esophagus [1,2,3,4,5,6,7,8,52,53,54,55,56].

A greater annual reduction in SC mortality rates was observed among White women and White men compared to Black women (1.20% vs. −1.12%) and Black men (−2.14% vs. −1.26%). Although these differences were not statistically significant at the 5% level, they may reflect racial inequalities in access to cancer diagnosis and treatment. Previous studies have shown that Black women are more likely to receive a late-stage diagnosis and experience delays in initiating treatment compared to White women, particularly among those with lower educational levels and greater reliance on the public healthcare system [20,21,22,24,57].

Regarding CRC, an increasing trend in incidence has been observed both in Brazil and globally [1,2,3,4,5,7,8,12,58,59,60,61]. Its etiology is strongly influenced by family history and behavioral factors. The primary contributors to the population attributable fraction for CRC incidence include unhealthy dietary habits (49.60%), such as diets low in calcium, milk, and fiber but high in red and processed meats, as well as alcohol consumption (15.20%), smoking (13.30%), a high body mass index (8.60%), elevated fasting glucose (7.80%), and low physical activity (3.50%) [61]. These findings align with patterns of population attributable risks documented in Brazil [62].

While developed countries have shown reductions or stabilization in CRC mortality rates, Brazil and other low- and middle-income countries continue to experience an upward trend [1,7,36,56,57,58,59]. This trend appears to be driven by population aging and lifestyle changes that have not been accompanied by effective screening programs, early detection efforts, or access to advanced therapeutic interventions [1,2,3,4,5,7,8,61,63,64,65,66,67]. Importantly, survival rates and temporal trends in CRC mortality are influenced by the socioeconomic development of the region of residence, the density of medical specialists, the availability of specialized healthcare networks, and the race/skin color of the patient [1,7,41,58,59].

We identified an increase in CRC mortality rates in both the White and Black populations. However, the rise in CRC mortality rates, coupled with a reduction in SC mortality, initially emerged in the White population. This pattern was also evident when analyzing the temporal trend of CRC according to the level of socioeconomic development and access to oncological care networks across different Brazilian regions. A previous study reported that the capitals of Brazil’s more developed regions, which possess robust oncological care networks, experienced reductions in SC mortality and increases in CRC mortality as early as the late 1990s. In contrast, reductions in SC mortality were only observed from 2014 onward in the capitals of regions characterized by greater socioeconomic vulnerability and weaker oncological care networks [41].

Research indicates that genetic and epigenetic differences may predispose Black individuals to a more pronounced inflammatory profile or greater susceptibility to *Helicobacter pylori* (*H. pylori*) infection compared to White individuals. These differences involve variations in pro-inflammatory cytokine polymorphisms, specific DNA methylation patterns, and potential mutations in DNA repair genes. Genetic, epigenetic, and immune response factors are hypothesized to interact, creating a microenvironment conducive to tumor development that may vary across population groups [52,68,69,70]. Notably, the prevalence of *H. pylori*—particularly CagA-positive strains—is significantly higher among low-income African Americans, which may contribute to their elevated risk [70].

In the context of colorectal cancer (CRC), studies suggest that Black individuals may exhibit distinct genetic and epigenetic alterations during carcinogenesis compared to White individuals. Molecular profiling studies highlight differences in the frequencies of mutations in the *KRAS*, *BRAF*, and *TP53* genes, as well as variations in DNA methylation patterns and other molecular changes. These distinct molecular predispositions are associated with neoplasms that demonstrate greater aggressive potential, often leading to diagnoses at more advanced stages [71,72,73].

However, it is crucial to emphasize that these biological differences between White and Black individuals remain inconclusive. They should not be interpreted as definitive explanations for racial disparities in cancer outcomes [68,69,70,71,72,73]. Instead, the role of structural racism must be recognized as a critical determinant of these disparities. Factors such as higher rates of *H. pylori* infection, greater consumption of starchy foods, and limited access to fresh produce are strongly associated with the increased risk of stomach cancer (SC) and colorectal cancer (CRC) among individuals from low socioeconomic and racial minority groups [58,74].

These social determinants of health underscore the necessity of addressing systemic inequities to reduce disparities in the incidence and mortality of SC and CRC. Our findings reveal a more pronounced increase in CRC mortality rates among Black individuals compared to Whites. Black women experienced an annual increase of 3.13%, compared to 1.67% among White women, while Black men exhibited a 4.42% annual increase compared to 2.63% among White men.

In Brazil, research has shown that Black race/skin color is associated with a higher likelihood of delayed CRC treatment, contributing to the observed mortality differences between White and Black populations [66]. Similar racial disparities in access to cancer treatment have been documented in the United States, where they impact disease staging at diagnosis [64]. It is noteworthy that CRC survival is closely linked to early detection, with survival rates nearing 90.0% for early-stage diagnoses but plummeting to 13.0% for advanced-stage diagnoses [67].

Early diagnosis and timely treatment are heavily dependent on access to healthcare services. In Brazil, medical specialists and specialized care are predominantly concentrated in the central urban areas of cities with higher socioeconomic development [16,17,19,36,58,63]. Within this context, it is crucial to highlight that the Black population primarily resides in regions with lower socioeconomic development, characterized by significant disparities in healthcare accessibility across all levels of care. These gaps increase the likelihood of advanced-stage cancer diagnoses among Black individuals [21,22,24,41,45,64,66].

### Strengths and Limitations of This Study

The findings of this study have some limitations. The first pertains to differences in the quality of death certification and the coverage of information systems between White and Black populations. To mitigate this issue, we corrected death records for misclassification using a methodology proposed by the WHO and previously applied in Brazilian studies. However, we could not address under-reporting, as no correction factors stratified by race/skin color were identified in the Brazilian literature. Given that under-reporting is not uniform between White and Black populations, we opted not to apply correction factors based solely on sex.

Another limitation is the inability to differentiate stomach cancer topographies (cardia and non-cardia), which exhibit distinct temporal patterns and variations by race/skin color. Evidence suggests that non-cardia stomach cancer incidence is higher among Black individuals compared to White individuals, reflecting the poorer living conditions experienced by the Black population [50,51,52,53,54]. This stratification was not possible because the Brazilian Mortality Information System does not distinguish cancers by topography.

Furthermore, Brazil’s significant intraregional differences in socioeconomic and racial inequalities were not addressed due to the lack of population estimates stratified by race/skin color, sex, and age group for Brazilian regions.

Despite these limitations, this study is among the first to analyze temporal trends in cancer mortality between White and Black populations in Brazil. The findings underscore disparities in the quality of death certification, the magnitude of mortality rates, and temporal trends between these groups, exposing systemic racial barriers. The observed racial disparities highlight the urgent need for targeted interventions to reduce these inequalities.

Brazil currently lacks screening programs for SC and CRC, emphasizing the necessity of measures that address modifiable risk factors, improve access to early diagnosis, and ensure timely treatment for both cancers. Preventive strategies should focus on addressing risk factors such as smoking, unhealthy diets, overweight, and obesity. Policies that facilitate access to natural foods—such as reducing their cost, promoting family farming, supporting state-funded urban gardens, and implementing stricter regulations on ultraprocessed food advertising—are essential. These efforts must be coupled with initiatives to reduce racial inequalities in access to diagnosis and timely treatment, prioritizing individuals at greater risk of developing these diseases.

## 5. Conclusions

We observed higher stomach cancer (SC) and colorectal cancer (CRC) mortality rates among White men and women, as well as a more pronounced reduction in SC mortality rates within this population. In contrast, there was a greater increase in CRC mortality rates among Black individuals. These findings underscore that, while Brazil has made progress in public health, racial inequalities remain a critical challenge. Addressing such disparities requires a multifaceted approach, including improving data collection, implementing inclusive policies, and ensuring equitable access to high-quality healthcare. These measures could help reduce the burden of these diseases and promote greater health equity.

## Figures and Tables

**Table 1:** Average standardized mortality rates * by four-year period, type of cancer, and race/skin color in women in Brazil (2000 to 2023).

Period	Stomach Cancer in Black Women		Stomach Cancer in White Women		*p*-Value ^a^
	Mortality Rate	Standard Deviation	Mortality Rate	Standard Deviation	
2000–2003	6.18	0.39	10.09	0.21	0.000
2004–2007	6.18	0.39	9.69	0.42	0.000
2008–2011	6.42	0.13	8.35	0.42	0.000
2012–2015	6.33	0.19	7.90	0.41	0.000
2016–2019	5.77	0.14	6.89	0.24	0.000
2020–2023	4.84	0.06	6.11	0.22	0.000
**Period**	**Colorectal Cancer in Black Women**		**Colorectal Cancer in White Women**		***p*** **-Value ^a^**
	**Mortality Rate**	**Standard Deviation**	**Mortality Rate**	**Standard Deviation**	
2000–2003	5.64	0.48	13.96	0.94	0.000
2004–2007	6.43	0.23	15.19	0.27	0.000
2008–2011	7.03	0.19	15.29	0.25	0.000
2012–2015	8.15	0.27	15.54	0.28	0.000
2016–2019	8.66	0.27	15.20	0.41	0.000
2020–2023	8.55	0.47	15.28	0.63	0.000

Note: * The standard population was proposed by the WHO (2000 to 2025) [37]. ^a^ Student’s *t*-test. Source: Mortality Information System (SIM/SUS)|Institute of Applied Economic Research (Instituto de Pesquisas Econômicas e Aplicadas—IPEA).

**Table 2:** Average standardized mortality rates * by four-year period, type of cancer, and race/skin color in men in Brazil (2000 to 2023).

Period	Stomach Cancer in Black Men		Stomach Cancer in White Men		*p*-Value ^a^
	Mortality Rate	Standard Deviation	Mortality Rate	Standard Deviation	
2000–2003	17.61	0.64	18.24	0.46	0.000
2004–2007	17.85	0.20	22.90	1.29	0.000
2008–2011	16.53	0.40	19.34	0.86	0.000
2012–2015	15.51	0.42	17.20	0.77	0.000
2016–2019	14.44	0.54	14.86	0.64	0.004
2020–2023	11.71	0.19	12.13	0.52	0.979
**Period**	**Colorectal Cancer in Black Men**		**Colorectal Cancer in White Men**		***p*** **-Value ^a^**
	**Mortality Rate**	**Standard Deviation**	**Mortality Rate**	**Standard Deviation**	
2000–2003	5.01	0.51	11.63	0.82	0.000
2004–2007	6.12	0.18	17.26	0.59	0.000
2008–2011	7.31	0.45	17.99	0.69	0.000
2012–2015	8.36	0.22	18.89	0.43	0.000
2016–2019	9.53	0.45	19.65	0.17	0.000
2020–2023	9.75	0.46	20.65	0.75	0.000

Note: * The standard population was proposed by WHO (2001) [37]. ^a^ Student’s *t*-test. Source: Mortality Information System (SIM/SUS)|Institute of Applied Economic Research (Instituto de Pesquisas Econômicas e Aplicadas—IPEA).

**Table 3:** Age-standardized average mortality rates * by four-year period, age group, type of cancer, and race/skin color in women in Brazil (2000 to 2023).

Stomach Cancer in Black Women—Mean (SD)				
Period	Age Groups (Years)			
	25 to 29	30 to 44	45 to 59	60 or more
2000–2003	0.36 (0.10)	1.42 (0.13)	5.46 (0.36)	26.33 (1.65)
2004–2007	0.42 (0.11)	1.67 (0.43)	5.62 (0.43)	27.13 (1.61)
2008–2011	0.55 (0.09)	1.63 (0.06)	5.64 (0.11)	23.98 (0.56)
2012–2015	0.46 (0.04)	1.77 (0.11)	5.73 (0.31)	22.54 (1.08)
2016–2019	0.47 (0.08)	1.76 (0.10)	5.24 (0.32)	19.87 (0.30)
2020–2023	0.47(0.21)	1.55 (0.15)	4.40 (0.23)	16.36 (0.33)
*p*-value ^a^	0.340	0.010	0.000	0.000
**Stomach Cancer in White Women—Mean (SD)**				
**Period**	**Age Groups (Years)**			
	**25 to 29**	**30 to 44**	**45 to 59**	**60 or more**
2000–2003	0.53 (0.10)	1.87 (0.11)	6.54 (0.18)	35.84(1.02)
2004–2007	0.56 (0.07)	1.99 (0.11)	6.11 (0.30)	34.24 (1.58)
2008–2011	0.45 (0.08)	1.82 (0.10)	6.05 (0.33)	28.28 (1.43)
2012–2015	0.56 (0.17)	1.91 (0.12)	5.71 (0.23)	26.33 (1.81)
2016–2019	0.58 (0.16)	1.84 (0.18)	5.24 (0.16)	22.28 (0.84)
2020–2023	0.47 (0.11)	1.86 (0.14)	4.46 (0.40)	19.63 (0.31)
*p*-value ^a^	0.59	0.51	0.000	0.000
**Colorectal Cancer in Black Women—Mean (SD)**				
**Period**	**Age Groups (Years)**			
	**25 to 29**	**30 to 44**	**45 to 59**	**60 or more**
2000–2003	0.32 (0.07)	1.19 (0.11)	4.69 (0.43)	18.45 (1.66)
2004–2007	0.37 (0.18)	1.36 (0.09)	5.72 (0.46)	20.50 (0.41)
2008–2011	0.53 (0.09)	1.49 (0.10)	6.29 (0.37)	22.27 (0.63)
2012–2015	0.61 (0.18)	1.83 (0.18)	7.34 (0.12)	25.61 (1.11)
2016–2019	0.50 (0.04)	1.84 (0.12)	7.41 (0.17)	27.9 (1.16)
2020–2023	0.48 (0.13)	1.91 (0.15)	7.55 (0.41)	27.18 (1.46)
*p*-value ^a^	0.023	0.000	0.000	0.000
**Colorectal Cancer in White Women—Mean (SD)**				
**Period**	**Age Groups (Years)**			
	**25 to 29**	**30 to 44**	**45 to 59**	**60 or more**
2000–2003	0.43 (0.11)	2.28 (0.20)	9.88 (1.21)	49.27 (2.62)
2004–2007	0.54 (0.10)	2.65 (0.14)	10.73 (0.35)	53.27 (1.06)
2008–2011	0.65 (0.12)	2.74 (0.12)	11.57 (0.54)	52.43 (0.30)
2012–2015	0.66 (0.09)	2.69 (0.09)	12 (0.19)	53.05 (1.41)
2016–2019	0.75 (0.17)	2.97 (0.17)	12.05 (0.16)	50.87 (1.87)
2020–2023	0.79 (0.25)	2.83(0.25)	10.95 (0.26)	52.97 (1.33)
*p*-value ^a^	0.0345	0.004	0.000	0.000

Note: * The standard population was proposed by WHO (2001) [42]. ^a^ Student’s *t*-test. SD—standard deviation. Source: Mortality Information System (SIM/SUS)|Institute of Applied Economic Research (Instituto de Pesquisas Econômicas e Aplicadas—IPEA).

**Table 4:** Age-standardized average mortality rates * by four-year period, age group, type of cancer, and race/skin color in men in Brazil (2000 to 2023).

Stomach Cancer in Black Men—Mean (SD)				
Period	Age Groups (Years)			
	25 to 29	30 to 44	45 to 59	60 or more
2000–2003	0.41 (0.18)	2.12 (0.07)	14.95 (0.62)	60.27 (2.92)
2004–2007	0.27 (0.06)	2.46 (0.14)	14.89 (0.24)	60.96 (1.45)
2008–2011	0.44 (0.07)	2.31 (0.10)	13.74 (0.21)	56.33 (2.29)
2012–2015	0.43 (0.06)	2.48 (0.17)	13.22 (0.41)	51.84 (2.25)
2016–2019	0.49 (0.08)	2.29 (0.10)	11.93 (0.64)	48.78 (2.10)
2020–2023	0.42(0.04)	2.02 (0.16)	9.44 (0.33)	39.57 (0.53)
*p*-value ^a^	0.071	0.000	0.000	0.000
**Stomach Cancer in White Men—Mean (SD)**				
**Period**	**Age Groups (Years)**			
	**25 to 29**	**30 to 44**	**45 to 59**	**60 or more**
2000–2003	0.42 (0.10)	2.50 (0.14)	18.42 (0.28)	87.91 (2.24)
2004–2007	0.53 (0.16)	2.38 (0.28)	16.68 (1.10)	82.72 (5.40)
2008–2011	0.51 (0.12)	1.96 (0.06)	14.34 (0.33)	69.59 (3.95)
2012–2015	0.47 (0.11)	1.82 (0.11)	13.25 (0.34)	61.11 (3.29)
2016–2019	0.55 (0.11)	1.57 (0.17)	10.75 (0.74)	53.58 (2.21)
2020–2023	0.48 (0.15)	1.64 (0.17)	8.48 (0.62)	43.46 (1.57)
*p*-value ^a^	0.757	0.000	0.000	0.000
**Colorectal Cancer in Black Men—Mean (SD)**				
**Period**	**Age Groups (Years)**			
	**25 to 29**	**30 to 44**	**45 to 59**	**60 or more**
2000–2003	0.42 (0.12)	1.06 (0.21)	3.25 (0.33)	10.85 (1.90)
2004–2007	0.53 (0.08)	1.35 (0.17)	4.39 (0.39)	15.04 (0.83)
2008–2011	0.59 (0.13)	1.70 (0.18)	5.97 (0.31)	19.84 (2.09)
2012–2015	0.63 (0.15)	2.14 (0.30)	7.95 (0.43)	24.94 (1.77)
2016–2019	0.65 (0.02)	2.76 (0.25)	9.89 (0.93)	31.22 (2.48)
2020–2023	0.98 (0.14)	3.11 (0.16)	10.93 (0.86)	35.03 (3.21)
*p*-value ^a^	0.000	0.000	0.000	0.000
**Colorectal Cancer in White Men—Mean (SD)**				
**Period**	**Age Groups (Years)**			
	**25 to 29**	**30 to 44**	**45 to 59**	**60 or more**
2000–2003	0.57 (0.13)	2.33 (0.25)	10.77 (0.41)	55.79 (3.43)
2004–2007	0.73 (0.07)	2.62 (0.12)	12.58 (0.29)	60.63 (2.77)
2008–2011	0.70 (0.14)	2.69 (0.17)	13.06 (0.81)	63.37 (2.15)
2012–2015	0.65 (0.11)	2.69 (0.23)	14.16 (0.43)	65.82 (1.89)
2016–2019	0.64 (0.10)	2.93 (0.20)	14.51 (0.29)	68.96 (1.03)
2020–2023	0.65 (0.11)	3.17 (0.23)	14.15 (0.57)	68.88 (3.03)
*p*-value ^a^	0.522	0.000	0.000	0.000

Note: * The standard population was proposed by WHO (2001) [42]. ^a^ ANOVA. SD = standard deviation. Source: Mortality Information System (SIM/SUS)|Institute of Applied Economic Research (Instituto de Pesquisas Econômicas e Aplicadas—IPEA).

**Table 5:** Annual Percent Change (APC) and its lower and upper limits for stomach and colorectal cancer in women by race/skin color in Brazil (2000 to 2023), estimated using Prais–Winsten regression.

Stomach Cancer in Black Women				
Age Group (Years)	APC	Lower Limit	Upper Limit	*p*-Value ^a^
25 to 29	0.35	−1.90	2.64	0.752
30 to 44	0.23	−0.70	1.18	0.609
45 to 59	−1.53	−2.48	−0.57	0.003
60 or more	−2.79	−3.46	−2.12	0.000
**Stomach Cancer in White Women**				
**Age Group (Years)**	**APC**	**Lower Limit**	**Upper Limit**	***p*** **-Value ^a^**
25 to 29	−0.27	−1.35	0.83	0.619
30 to 44	−0.31	−0.59	−0.03	0.030
45 to 59	−1.84	−2.28	−1.41	0.000
60 or more	−3.17	−3.54	−2.80	0.000
**Colorectal Cancer in Black Women**				
**Age Group (Years)**	**APC**	**Lower Limit**	**Upper Limit**	***p*** **-Value ^a^**
25 to 29	2.68	−0.04	5.47	0.054
30 to 44	2.35	1.67	3.02	0.000
45 to 59	1.84	0.90	2.78	0.001
60 or more	1.91	1.36	2.46	0.000
**Colorectal Cancer in White Women**				
**Age Group (Years)**	**APC**	**Lower Limit**	**Upper Limit**	***p*** **-Value ^a^**
25 to 29	3.30	2.13	4.48	0.000
30 to 44	1.06	0.48	1.65	0.001
45 to 59	0.28	−0.36	0.93	0.367
60 or more	0.29	−0.09	0.67	0.131

Note: ^a^ Student’s *t*-test with n−2 degrees of freedom.

**Table 6:** Annual Percent Change (APC) and its lower and upper limits for stomach and colorectal cancer in men by race/skin color in Brazil (2000 to 2023), estimated using Prais–Winsten regression.

Stomach Cancer in Black Men				
Age Group (Years)	APC	Lower Limit	Upper Limit	*p*-Value ^a^
25 to 29	0.68	−1.45	2.86	0.52
30 to 44	−0.58	−1.77	0.63	0.33
45 to 59	−2.84	−3.75	−1.91	0.00
60 or more	−2.47	−2.98	−1.96	0.00
**Stomach Cancer in White Men**				
**Age Group (Years)**	**APC**	**Lower Limit**	**Upper Limit**	***p*** **-Value ^a^**
25 to 29	0.31	−0.95	1.59	0.61
30 to 44	−2.42	−3.18	−1.66	0.00
45 to 59	−4.14	−4.81	−3.48	0.00
60 or more	−3.73	−4.02	−3.44	0.00
**Colorectal Cancer in Black Men**				
**Age Group (Years)**	**APC**	**Lower Limit**	**Upper Limit**	***p*** **-value ^a^**
25 to 29	1.31	0.25	2.38	0.02
30 to 44	2.65	2.25	3.05	0.00
45 to 59	2.90	1.95	3.86	0.00
60 or more	3.36	2.66	4.06	0.00
**Colorectal Cancer in White Men**				
**Age Group (Years)**	**APC**	**Lower Limit**	**Upper Limit**	***p*** **-Value ^a^**
25 to 29	−0.06	−0.95	0.84	0.895
30 to 44	1.18	0.67	1.69	0.000
45 to 59	1.02	0.32	1.71	0.006
60 or more	0.96	0.71	1.20	0.000

Note: ^a^ Student’s *t*-test with n−2 degrees of freedom.

## Data Availability

The data are contained in the article and Supplementary Materials and in the zenodo repository (https://doi.org/10.5281/zenodo.14727649, accessed on 15 November 2024).

## Supplementary Materials

The following supporting information can be downloaded at https://www.mdpi.com/article/10.3390/ijerph22020208/s1: Table S1: Number of uncorrected and corrected deaths and uncorrected and corrected standardized mortality rates per 100,000 for stomach cancer and colon and rectal cancer in Brazil (2000 to 2023). Table S2: Average Annual Percent Change for stomach and colorectal cancer by sex and race/skin color in Brazil (2000 to 2023), estimated using Prais–Winsten regression.
