# Supplementary material for: Temporal Trends in Stomach and Colorectal Cancer Mortality by Racial Groups in Brazil (2000–2023): A Longitudinal Ecological Study
Source: Int J Environ Res Public Health. 2025 Jan 31;22(2):208. doi: 10.3390/ijerph22020208 (PMC11855464; doi:10.3390/ijerph22020208)
Supplement: Supplementary file 1 [file ijerph-22-00208-s001.zip › S1 Table.pdf]

S1 Table. Number of uncorrected and corrected deaths, uncorrected and corrected standardized mortality rates per 100,000, for stomach cancer and colon and rectal cancer, Brazil (2000 to 2023), Brazil, 2024.

**Stomach cancer in Black women**	**N**	**Standardized mortality rates**	**Δ%**
number of deaths without correction	44029	5.75	12.52
number of corrected deaths	49617	6.47	
**Stomach cancer in White women**	**N**	**Standardized mortality rates**	**%**
number of deaths without correction	61974	7.13	9.96
number of corrected deaths	68530	7.84	
**Colorectal cancer in Black women**	**N**	**Standardized mortality rates**	**Δ%**
number of deaths without correction	52540	6.97	11.76
number of corrected deaths	58715	7.79	
**Colorectal cancer in White women**	**N**	**Standardized mortality rates**	**Δ%**
number of deaths without correction	120503	13.64	10.85
number of corrected deaths	133209	15.12	
**Stomach cancer in Black men**	**N**	**Standardized mortality rates**	**Δ%**
number of deaths without correction	83581	12.76	16.37
number of corrected deaths	97328	14.85	
**Stomach cancer in White men**	**N**	**Standardized mortality rates**	**%**
number of deaths without correction	81192	16.04	9.66
number of corrected deaths	89498	17.59	
**Colorectal cancer in Black men**	**N**	**Standardized mortality rates**	**Δ%**
number of deaths without correction	49188	7.27	14.71
number of corrected deaths	55186	8.34	
**Colorectal cancer in White men**	**N**	**Standardized mortality rates**	**%**
number of deaths without correction	113612	16.74	10.69
number of corrected deaths	125022	18.53	

Note: *The standard population was proposed by WHO (2005 to 2025)l[37].Source: Mortality Information System (SIM/SUS) | Institute of Applied Economic Research (Instituto de Pesquisas Econômicas e Aplicadas -IPEA).
