# Supplementary material for: Temporal Trends in Stomach and Colorectal Cancer Mortality by Racial Groups in Brazil (2000–2023): A Longitudinal Ecological Study
Source: Int J Environ Res Public Health. 2025 Jan 31;22(2):208. doi: 10.3390/ijerph22020208 (PMC11855464; doi:10.3390/ijerph22020208)
Supplement: Supplementary file 1 [file ijerph-22-00208-s001.zip › S2 Table.pdf]

S2 Table. Average Annual Percent Change for Stomach and Colorectal Cancer, by Sex and Race/Skin Color, Brazil (2000 to 2023), Estimated Using Prais-Winsten Regression.

Type of cancer	APC (%)	CI Lower (%)	CI Upper (%)	p-value[a]
Stomach Cancer in White Men	-2.14	-2.50	-1.77	0.004
Stomach Cancer in Black Men	-1.26	-2.05	-0.46	0.000
Colorectal cancer in White men	2.63	2.31	2.95	0.000
Colorectal cancer in Black men	4.42	3.73	5.11	0.000
Stomach cancer in Black women	-1.12	-1.94	-0.29	0.011
Stomach cancer in White women	-1.20	-1.52	-0.88	0.000
Colorectal cancer in Black women	3.13	2.31	3.95	0.000
Colorectal cancer White woman	1.67	1.39	1.96	0.000

Note: [a]t -Student with n−2 degrees of freedom
